# The relationship of recombination rate, genome structure, and patterns of molecular evolution across angiosperms

**DOI:** 10.1186/s12862-015-0473-3

**Published:** 2015-09-16

**Authors:** George P. Tiley, Gordon Burleigh

**Affiliations:** Department of Biology, University of Florida, Bartram-Carr Hall, Gainesville, FL 32611 USA

## Abstract

**Background:**

Although homologous recombination affects the efficacy of selection in populations, the pattern of recombination rate evolution and its effects on genome evolution across plants are largely unknown. Recombination can reduce genome size by enabling the removal of LTR retrotransposons, alter codon usage by GC biased gene conversion, contribute to complex histories of gene duplication and loss through tandem duplication, and enhance purifying selection on genes. Therefore, variation in recombination rate across species may explain some of the variation in genomic architecture as well as rates of molecular evolution. We used phylogenetic comparative methods to investigate the evolution of global meiotic recombination rate in angiosperms and its effects on genome architecture and selection at the molecular level using genetic maps and genome sequences from thirty angiosperm species.

**Results:**

Recombination rate is negatively correlated with genome size, which is likely caused by the removal of LTR retrotransposons. After correcting recombination rates for euchromatin content, we also found an association between global recombination rate and average gene family size. This suggests a role for recombination in the preservation of duplicate genes or expansion of gene families. An analysis of the correlation between the ratio of nonsynonymous to synonymous substitution rates (*dN/dS*) and recombination rate in 3748 genes indicates that higher recombination rates are associated with an increased efficacy of purifying selection, suggesting that global recombination rates affect variation in rates of molecular evolution across distantly related angiosperm species, not just between populations. We also identified shifts in *dN/dS* for recombination proteins that are associated with shifts in global recombination rate across our sample of angiosperms.

**Conclusions:**

Although our analyses only reveal correlations, not mechanisms, and do not include potential covariates of recombination rate, like effective population size, they suggest that global recombination rates may play an important role in shaping the macroevolutionary patterns of gene and genome evolution in plants. Interspecific recombination rate variation is tightly correlated with genome size as well as variation in overall LTR retrotransposon abundances. Recombination may shape gene-to-gene variation in *dN/dS* between species, which might impact the overall gene duplication and loss rates.

**Electronic supplementary material:**

The online version of this article (doi:10.1186/s12862-015-0473-3) contains supplementary material, which is available to authorized users.

## Background

Meiotic recombination has been a topic of interest in evolutionary biology since Fisher first addressed the effects of linkage on substitutions in a population [1], yet the macroevolutionary consequences of recombination on plant genomes are still poorly understood. Comparative studies of the effects of recombination rate on genome architecture and sequence evolution across distantly related species require both whole genome sequences and data-intensive estimates of recombination rates [2–4]. Recent genome sequencing and genetic mapping efforts, which provide physical measurements of genome size and map length, make studies of global recombination rate possible in plants. In this study, we take advantage of these new data to explore the relationship between recombination rate, genome structure, and patterns of molecular evolution throughout angiosperms in order to better characterize the broad macroevolutionary patterns of recombination rate variation and its possible consequences for genome evolution.

Recombination affects both genome architecture and evolutionary rates. Lynch [5] showed that generation scaled global recombination rate (centimorgans/basepairs/generation) decreases as species genome size increases in unicellular eukaryotes, invertebrates, vertebrates, and land plants. Similarly, in plants, Cavalier-Smith [6] proposed that the recombination rate is higher in smaller angiosperm genomes than in larger genomes. Rees and Durrant [7] corroborated this result in a study of the genera *Lathyrus, Lolium, and Petunia* and by Narayan and McIntyre [8] in *Lathyrus*. Both of these studies estimated nuclear genome size in picograms per haploid genome (C-values) and recombination rates based on observable chiasma from pachytene chromosomes. Typically, one observable chiasma is expected per chromosome arm for segregation to proceed normally. However, the number of crossovers per chromosome arm is variable [9], and Ross-Ibarra [10] demonstrated a positive correlation between genome size and the number of chiasmata per chromosome arm across 279 angiosperm species from 22 families.

One potential mechanism for a negative association between global recombination rate and genome size is that recombination either deletes LTRs by chance or it facilitates selection against transposable element insertions [11]. Much of the genome size variation in flowering plants can be attributed to changes in repetitive element content, and specifically long terminal repeat (LTR) retrotransposons [12, 13]. The loss of LTR retrotransposon content can occur through unequal homologous recombination [14]. Thus, lineages with higher recombination rates are expected to have lower LTR retrotransposon content, and hence smaller genomes [15], as well as higher gene densities. It is not clear if recombination preferentially removes specific families of LTR retrotransposons; since LTR retrotransposons are removed by unequal crossing over due to high sequence identity, we might expect all LTR retrotransposon families to be removed equally. Additionally, all LTR retrotransposon families appear to have similar life histories in rice [16], suggesting LTR retrotransposons vary only in abundance. Regions of the genome with little or no recombination (i.e., mainly heterochromatin during crossing over) have longer transposable elements and lower gene density when compared to regions with frequent recombination [17], and recombination rate and gene density are positively correlated in the genomes of maize, rice, wheat, and *Arabidopsis thaliana* [18–21].

Recombination rate also has been linked to the GC content and codon usage bias of genes due to GC biased gene conversion [22]. Although DNA mismatch repair during crossover resolution can be GC biased [23], the strength of selection for a site and the effects of linkage alone can alter local codon usage landscapes [24]. GC biased gene conversion drives a positive relationship between local recombination rate and codon bias within the *Caenorhabditis elegans* and *Drosophila melanogaster* genomes [25]. There is also a positive correlation between GC content and local recombination rate across mammals [26] and within humans [27, 28], which may indicate the strength of GC biased gene conversion. However, the relationship between recombination and compositional biases in angiosperms is unclear. Local recombination rate is weakly negatively correlated with GC content in *Medicago truncatula* [29], but not within self-fertilizing populations of *Arabidopsis thaliana*, likely due to reduced heterozygosity [30]. Correlations between recombination rate and GC content appear to be a feature of exclusively outcrossing species [31]. Despite the lack of an obvious relationship between recombination rate and GC content across most plant species [30], there is evidence that GC biased gene conversion is occurring in some lineages. For instance, individual gene families in grasses show evidence of nucleotide composition biases and gene conversion [32].

Within populations, recombination can create favorable combinations of alleles that may have a selective advantage in future generations, while linkage between sites may reduce the efficacy of selection [33, 34], a phenomenon known as Hill-Robertson effects [35]. Hill-Robertson effects include hitchhiking [36], fixation of sites linked to a beneficial mutation, and background selection [37] or loss of variation linked to a deleterious mutation, which lead to reduced effective population size for a genomic region with a low recombination rate. Recombination rate is negatively associated with the ratio of nonsynonymous to synonymous substitution rates (*dN/dS*) within genomes and positively correlated with *dS* in model organisms such as *Drosophila melanogaster* [38]. However, evidence of Hill-Robertson effects is typically weaker in plant genomes [39, 40], even when considering variation in life history traits [41]. Moreover, it is not clear if the effects of recombination rates are pervasive over long evolutionary time periods, since recombination landscapes can vary over time [42, 43] and across populations [44], and if recombination rate is associated with *dN/dS* between species. For example, there was no association between recombination rate and rates of molecular evolution in comparisons between *Arabidopsis thaliana* and *A. lyrata* [45].

Both tandemly duplicated genes [46–48] and dispersed duplicates [48] are more prevalent in regions of the genome with high recombination. The long-term survival of duplicate genes may be enhanced by purifying selection, which is more effective in regions of high recombination [36]. The probability of subfunctionalization or neofunctionalization of a duplicate gene increases with recombination rate [49, 50], and once the new gene copy has reached fixation, the probability of the duplicate gene’s survival also increases with recombination rate [51]. Given these expectations and observations of more duplicate genes in regions of high recombination, we hypothesize that species with higher global recombination rates may have more duplicate genes, resulting in larger gene families.

Here we make a first attempt to characterize the potential macroevolutionary role of recombination rate in shaping plant genomes. We examine correlates of global recombination rate across thirty phylogenetically diverse angiosperm species, with respect to genome architecture, compositional biases, and *dN/dS* in 3748 single-copy nuclear genes.

## Methods

### Recombination Rate Estimates and Genome Architecture

**Fig. 1:**
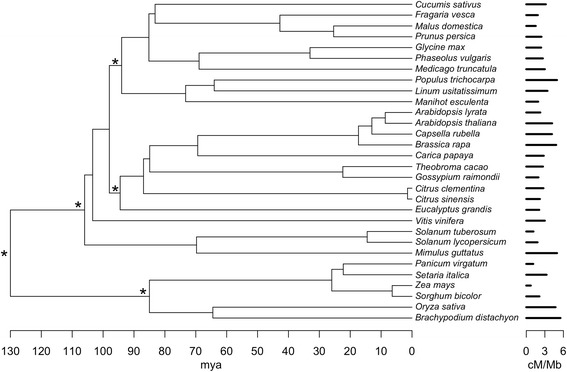
Ultrametric species tree of taxa used for comparative analyses. Divergence times were estimated based on molecular branch lengths and fossil calibrations using r8s. Asterisks denote nodes with fossil calibrations. The distribution of recombination rate (*cM*/*Mb*) is given along the tips

We assembled data for thirty angiosperm species with sequenced genomes and linkage maps from the primary literature (Fig. 1; Additional file 1: Table S1; Additional file 2). Only genetic maps where the numbers of linkage groups correspond to the haploid chromosome number were used to estimate global recombination rate, and we used multiple maps for each species and calculated recombination rate from average map lengths (Additional file 1: Table S2). We corrected map lengths for each species for marker density using method 4 of Chakravarti et al. [52], as implemented by Hall and Willis [53] and Dumont and Payseur [54]. Global recombination rate was measured by taking the corrected map length divided by the genome size in megabases (*cM*/*Mb*), where genome size is the total mapped and unmapped scaffold assembly size. Genome sizes were obtained from primary literature and early release statistics available on Phytozome (Table 1; citations are provided in Additional file 1: Table S1).

**Table 1:** All trait data used in the study are displayed. When trait data was unavailable for certain species NA is used. Citations are provided in the supplementary material

Species	cM/Mb	Euchromatin cM/Mb	Genome size (MB)	Proportion of LTRs	Proportion copia	Proportion gypsy	Gene density	Average gene family size
*Arabidopsis lyrata*	2.26	2.87	207.0	0.200	NA	NA	157.83	0.768
*Arabidopsis thaliana*	4.17	4.27	125.0	0.040	NA	NA	248.91	1.209
*Brachypodium distachyon*	5.52	NA	272.0	0.214	0.049	0.161	93.87	0.952
*Brassica rapa*	4.83	5.10	283.8	0.271	NA	NA	144.99	0.790
*Capsella rubella*	4.15	NA	134.8	0.094	NA	NA	196.74	0.751
*Carica papaya*	2.82	3.22	372.0	0.333	0.055	0.278	66.52	0.712
*Citrus clementina*	2.77	3.31	301.4	0.199	0.079	0.120	81.40	0.787
*Citrus sinensis*	2.19	2.62	320.5	0.153	0.071	0.082	91.87	0.784
*Cucumis sativus*	3.15	3.41	243.5	0.104	0.054	0.038	109.58	0.749
*Eucalyptus grandis*	2.09	NA	691.0	0.219	NA	NA	52.64	0.790
*Fragaria vesca*	1.86	NA	240.0	0.160	NA	NA	104.38	1.924
*Glycine max*	2.40	3.38	1060.0	0.430	0.130	0.300	43.80	0.867
*Gossypium raimondii*	1.96	3.97	761.4	0.449	0.338	0.111	53.82	0.821
*Linum usitatissimum*	3.42	NA	318.3	0.184	NA	NA	136.30	0.823
*Malus domestica*	1.52	NA	742.3	0.307	0.055	0.252	77.31	0.845
*Manihot esculenta*	1.91	3.03	760.0	0.111	NA	NA	40.35	0.811
*Medicago truncatula*	3.00	3.35	257.6	0.242	0.410	0.057	171.33	0.708
*Mimulus guttatus*	4.96	NA	321.7	0.200	0.100	0.100	87.47	0.790
*Oryza sativa*	4.73	5.41	372.0	0.235	0.025	0.120	114.66	1.084
*Panicum virgatum*	1.11	NA	1358.0	NA	NA	NA	72.17	0.805
*Phaseolus vulgaris*	2.67	2.78	521.1	0.367	0.094	0.251	52.19	0.794
*Populus trichocarpa*	4.96	6.73	485.0	0.065	0.016	0.049	94.13	1.431
*Prunus persica*	2.45	2.54	227.3	0.186	0.086	0.100	122.53	0.813
*Setaria italica*	3.28	NA	405.7	0.250	NA	NA	87.43	0.736
*Solanum lycopersicum*	1.81	7.50	760.0	0.618	0.063	0.197	45.69	2.156
*Solanum tuberosum*	1.15	1.63	727.0	0.522	0.038	0.152	48.15	1.232
*Sorghum bicolor*	2.12	4.02	738.5	0.544	0.052	0.190	37.43	1.464
*Theobroma cacao*	2.69	NA	326.9	0.160	0.070	0.090	88.09	1.537
*Vitis vinifera*	2.99	NA	487.1	0.145	0.084	0.032	68.80	0.760
*Zea mays*	0.72	1.24	2066.4	0.751	0.218	0.377	15.75	0.738

Our analyses assume that recombination rates estimated from genetic maps covary with rates of unequal crossing over. The rates of allelic homologous recombination appears to be a reasonable indicator of the rates of non-allelic crossing over in *Saccharomyces cerevisiae* [55–58], but this remains to be broadly shown in plants. We might not expect allelic crossing over to always be a reasonable predictor of non-allelic crossing over though, since non-allelic crossing over is dependent on genome spatial complexity [58].

Estimates of genome size in megabases may contain error due to the genome assembly. Therefore, we also calculated global recombination rates using C-values (cM/pg) as the estimates of genome size. C-values were taken from the Kew C-Value Database (http://data.kew.org/cvalues/; last accessed 22 August 2014; Additional file 1: Table S3). Some species used in this study can have different ploidy levels, resulting in multiple, distinct C-values (Additional file 1: Table S3). To test the effects of the different ploidy levels on the correlations between recombination rate and genome size, we generated 100 datasets by randomly selecting a single C-value for each species and performed phylogenetically corrected correlations for both cM/pg and pg and cM/pg and Mb. We performed a meta-analysis of the correlation coefficients with fixed effects using the R package metacor [59].

In addition to genome size, we also looked at the relationship between recombination rate and genome compactness, defined as the genome size over the haploid chromosome number. Recombination rates should be higher on shorter chromosome arms, since at least one crossover per chromosome arm is expected [9]. Chromosome size has been used as an indirect measure of recombination rate variation within a genome [31], so we tested if the overall genome compactness (genome size/haploid chromosome number) was correlated with global recombination rate. We also tested if haploid chromosome number was correlated with global recombination rate.

Global recombination rates estimated using the map length over the total genome size might not be directly comparable between species because recombination generally occurs in euchromatic regions of chromosomes during meiosis (e.g., [60]). For example, 97, 98, and 95 % of the genetic maps correspond to euchromatin in *Sorgum bicolor*, *Oryza sativa*, and *Zea mays* respectively [61]. The amount of the genome that is euchromatic during crossing-over can vary greatly between species. Thus, we also estimated a corrected recombination rate based on the euchromatic proportion of the genome for the 19 species (Table 1) in which fluorescence *in situ* hybridization or other analyses of pachytene chromosomes were performed to differentiate the chromosomal characteristics during meiosis. We used relative percentages of heterochromatin from the literature and subtracted that from the genome assembly size for each species (citations for differential chromatin studies are in Additional file 1: Table S1). We assumed 95 % of the genetic map lies in the euchromatic portion of the genome during crossing over. Thus, the euchromatin corrected recombination rate is equal to the total scaffold size minus the estimated percentage of heterochromatin in megabases over 95 % of the marker-density corrected map length.

For the 29 species with published genomes and available transposable element data, we obtained the proportion of the genome consisting of all LTR retrotransposons (Table 1). This was used to calculate genome size without LTR retrotransposons by subtracting the percent content of LTR retrotransposons from the total genome size. Estimating genome size without LTR retrotransposon content was done to address if an association between recombination rate and genome size can be explained by LTR retrotransposon content alone. Detailed transposable element classification was available for 20 species, which allowed us to investigate if relationships between recombination rate and LTR retrotransposon content could be explained by the proportion of *copia* or *gypsy* superfamilies. The *copia* and *gypsy* superfamilies were selected because they are generally the most abundant LTR retrotransposon classes and constitute most of the variation in LTR retrotransposons in plants.

Finally, gene density was obtained from the literature or early release statistics by dividing the number of predicted genes by the genome size. These data were available for all 30 species used in the study (Table 1).

### Sequence Data and Genome Content

Gene families for the 30 angiosperms with recombination rate data were downloaded from Phytozome v9.1 (www.phytozome.net, Last accessed 29 September 2013). Gene sequences were clustered into families based on reciprocal BLASTP distances, with full details described in Goodstein et al. [62]; clusters are provided by JGI through Phytozome using the BioMart tool. We translated the nucleotide sequences from Phytozome into amino acids and then aligned the amino acid sequences with MUSCLE 3.8.31 [63]. We obtained in-frame nucleotide alignments by mapping the codons to the aligned amino acid sequences using in-house Perl scripts. Perl scripts were also used to calculate GC content at 3^rd^ position 4-fold degenerate sites (3GC^S^) and codon bias, measured as effective number of codons (ENC; [64]), for each sequence for each gene family. We used ENC to measure codon bias because it is not biased by functional constraints of amino acid composition or gene length [64, 65]. We were interested in 3GC^S^ because we wanted to test if GC biased gene conversion is detectable throughout the genome in plants. If GC biased gene conversion is generally occurring, then we would expect a stronger bias in 3GC^S^ for genomes with higher recombination rates. Additionally, we calculated the average gene family size from the number of genes in each gene family to test if recombination facilitates gene duplication or the preservation of duplicate genes. Only gene families that spanned the root of the tree in Fig. 1 were used; this included 11,250 of the 12,748 Phytozome gene families.

### Species Tree for Comparative Analyses

For the phylogenetically informed analyses, we used a species tree (Fig. 1) with a topology that corresponds to our current understanding of angiosperm phylogeny between species (www.phytozome.net; e.g., [66]). While accounting for phylogenetic uncertainty is important in many studies, the relationships of the 30 taxa used here are mostly well established, and it is computationally prohibitive to repeat some analyses in this study using a distribution of trees. Full chloroplast genomes were not available for all species. Therefore, molecular branch lengths were estimated from an alignment of *matK* sequences (aligned length of 2036 bp) downloaded from Genbank (http://www.ncbi.nlm.nih.gov) using the GTR Γ model implemented in HYPHY 2.1.2.28 [67]. *MatK* is noted for providing reasonable topology and branch length estimates across angiosperms [68]. We transformed the branch lengths to make them ultrametric using penalized likelihood in r8s [69]. *Amborella trichopoda* was used as the outgroup, and the age of the most recent common ancestor of angiosperms was fixed to 150 million years ago (mya). Minimum age constraints were placed on Poaceae (65 mya; [70, 71]), Fabidae (94 mya; [71, 72]), and Malvidae (94 mya; [71]). Maximum age constraints were also placed at the most recent common ancestor of core Eudicots (124 mya; [71, 73, 74]), and Eudicots and Monocots (130 mya; [71]). The best smoothing parameter for the penalized likelihood analysis, 3200, was determined by cross validation. For the phylogenetic independent contrast analyses, to make comparisons consistent with the assumption that a contrast’s mean is independent of its standard deviation [75], contrasts were analyzed using the PDAP package in MESQUITE [76, 77] and a base-10 logarithmic transformation was performed on the ultrametric branch lengths.

### Phylogenetic structure

We calculated Blomberg’s *K* [78, 79] to test for a phylogenetic signal for recombination rate, genome size, LTR retrotransposon proportions, gene density, gene family size, global composition biases, genome compactness, and haploid chromosome number under a Brownian motion model of evolution (Additional file 1: Table S4). Blomberg’s *K* is the ratio of the mean squared error (MSE) of trait values on the tips of the phylogeny and the MSE expected under Brownian motion. A value of *K* greater than one implies phylogenetic clustering of traits, and values less than one are consistent with a lack of structure or overdispersion. The significance of a calculated *K* value was determined by 999 random permutations of the tips on the phylogeny performed using the R package picante [80].

### Phylogenetic Independent Contrasts

To account for the covariance of character states due to the shared phylogenetic history of different species, we performed phylogenetic independent contrasts (PICs; [81]) to examine the correlation between recombination rate and various characteristics of the genome. All PICs were obtained using the R package APE 3.0-11 [82]. We used Pearson’s correlation coefficient to test for associations of the phylogenetic independent contrast values of traits. All correlation analyses were performed in R [83]. All contrast analyses were performed for both raw recombination rate and euchromatin corrected recombination rate. We examined the relationship between global recombination rate and total genome size, genome size without LTR retrotransposon content, the relative abundance of LTR retrotransposons, gene density, and gene family size. We also calculated the power for all correlation analyses using the R package pwr [84], which uses the calculations provided by Cohen [85].

### Selection analyses

Single-copy gene families from Phytozome with sequences from more than ten species were evaluated to determine the relationship between recombination rate and *dN/dS* with COEVOL [86], using partial correlations that control for covariation in *dS*. This was done to investigate differences in levels of selection in genomes with variable rates of recombination. Since the method of Lartillot and Poujol [86] relies on a given species tree, only single-copy gene families containing no obvious paralogs were analyzed. To maximize taxonomic sampling, only raw recombination rates were used. *dN* and *dS* were optimized along the species tree, and ultrametric branch lengths for the species tree were fixed for performing contrasts. This was done to help the convergence of chains and reduce computational complexity. Two chains were run up to 72 hours, each with geodesic averaging of traits, and convergence of chains was determined by an effective sample size (ESS) greater than 300 for all parameters, with the exception of the ancestral state at the root, where the sampling state is especially difficult over large evolutionary time periods. An ESS of 50 was used for the root ancestral state, which yields qualitatively similar runs [86]. Parameter estimates for the largest alignments had converged by 72 hours, and parameter estimates that did not converge by this point likely indicated uncertainty in the data, possibly due to alignment, clustering, or annotation. Gene families with chains that did not converge were not used in the independent contrast analyses. For chains that did converge, 25 % of the chain was discarded as burn-in, and partial correlation coefficients for recombination rate and *dN/dS* were calculated for independent contrasts in COEVOL.

## Results

### Phylogenetic Structure of Traits

We first asked if there was a phylogenetic signal for recombination rate as well as several features of genome architecture. Global recombination rate, euchromatin corrected recombination rate, genome size, the proportion of the genome that consists of LTR retrotransposons, and average gene family size do not deviate significantly from Brownian motion. Both global ENC and GC3^S^ have significant phylogenetic structure, meaning that trait values are more similar amongst closely related species. Genome size without LTR retrotransposons, gene density, genome compactness, and haploid chromosome numbers are phylogenetically overdispersed, such that there is more variation than expected under Brownian motion (Additional file 1: Table S4).

### Recombination Rate and Genome Architecture

In the next analyses, we evaluate if there is a correlation between global recombination rate and genome size, LTR retrotransposon content, and gene density. If recombination enables the elimination of LTR retrotransposons, we may expect negative correlations between recombination rate and genome size and LTR retrotransposon content and a positive correlation between recombination rate and gene density.

**Fig. 2:**
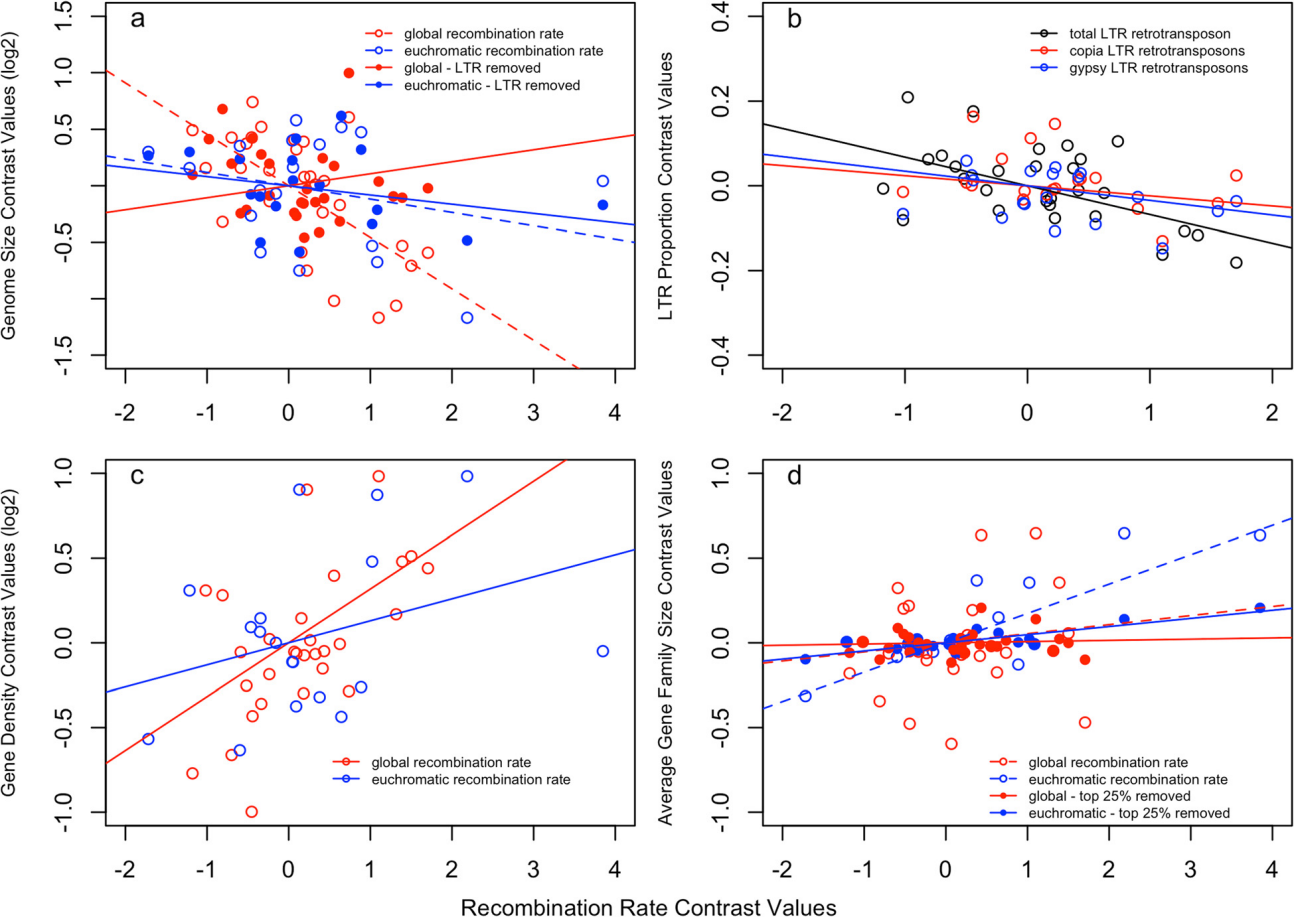
**a** Phylogenetic independent contrasts for both global recombination rate and euchromatin corrected recombination rate with genome size and genome size without LTR retrotransposons. Size estimates were log_2_ transformed for normality to satisfy assumptions of phylogenetic independent contrasts and parametric correlations. **b** Global recombination rate plotted against total LTR retrotransposon content as well as the proportions of *copia* and *gypsy* superfamilies. **c** Gene density is based on gene number and genome size estimate from literature. **d** Plots of recombination rate vs average gene family size based on both the 11,250 families that span the root as well as smallest 75 % of gene families. The best-fit linear regression is given for each contrast plot

Phylogenetic independent contrast analyses show a strong negative correlation between the global recombination rate and genome size (Fig. 2a; *r* = −0.65, *p* < 0.001). However, this strong negative correlation breaks down after removing the LTR retrotransposon content (Fig. 2a; *r* = 0.15, *p* = 0.460). Plant genome size is strongly, positively correlated with total LTR retrotransposon content (*r* = 0.72, *p* < 0.001), and global recombination rate is negatively associated with LTR retrotransposon content (Fig. 2b; *r* = −0.56, *p* = 0.002). The correlation between recombination rate and LTR retrotransposon content is not biased towards either the *gypsy* (*r* = −0.31, *p* = 0.200) or *copia* (*r* = −.34, *p* = 0.160) superfamilies. Recombination rate also is positively correlated with gene density (Fig. 2c; *r* = 0.57, *p* = 0.001). There is no detectable correlation between the global recombination rate and the average gene family size (Fig. 2d; *r* = 0.16, *p* = 0.410).

The global recombination rate and euchromatin corrected recombination rate are correlated (*r* = 0.69, *p* = 0.001), but euchromatin corrected recombination rate is not significantly correlated with most of the genomic traits including genome size (Fig. 2a; *r* = −0.28, *p* = 0.265), genome size without LTR retrotransposon content (Fig. 2a; *r* = −0.31, *p* = 0.212), LTR retrotransposon content (*r* = −0.11, *p* = 0.673) including both the *gypsy* (*r* = −0.08, *p* = 0.789) and *copia* (*r* = −0.23, *p* = 0.435) contributions, and gene density (Fig. 2c; *r* = 0.34, *p* = 0.173). However, there is a strong positive correlation between euchromatin corrected recombination rate and average gene family size (Fig. 2d; *r* = 0.82, *p* < 0.001). Global recombination rate is not correlated with genome compactness or haploid chromosome number (Additional file 3: Figure S1). However, euchromatin corrected recombination rate is negatively correlated with genome compactness (Additional file 3: Figure S1; *r =* −0.63, *p* = 0.027).

The lack of significant correlations using euchromatin corrected recombination rate, but not global recombination rate, possibly due to the smaller sample size. For example, we have power of 0.98 to refute the null hypothesis for raw recombination rate and genome size with a sample of 30, but only power of 0.32 to refute the null hypothesis for euchromatin corrected recombination rate and genome size with a sample of 19. To have power of 0.8 for correlations of PICs and sample size of 19, the correlation coefficient would need to be 0.62. Some of the differences may be biological as well, considering the correlation coefficient between global recombination rate and genome size is −0.57 (*p* = 0.013) for the same sample of 19 taxa.

### Addressing Uncertainty in Genomic Architecture

Next, we addressed some possible sources of uncertainty and error in the correlation analyses. The heterogeneous sources of data may produce uncertainty or error in our results. For example, the difficulty of assembling repetitive DNA in a genome sequence can lead to underestimates of genome size [87] and inaccurate estimates of transposable element content. Although this may introduce error into the estimates of genome size or transposable element content, we do not think it is biasing the analyses. Correlations between global recombination rates estimated with C-values, which are not affected by the ability to assemble repetitive DNA, and genome size in Mb yield similar results (Additional file 1: Table S5 and Table S6).

An additional concern when investigating plant genome size is the history of polyploidy. Not all taxa examined in this study are diploid, and they have different histories of lineage-specific polyploidy events. This concern was addressed by Ross-Ibarra [10], who demonstrated that including or excluding polyploids from analyses had little to no effect on the relationship between recombination rate and genome size. Our results also suggest that polyploidy does not influence the general relationship between recombination rate and genome size. Analysis of C-values for non-heterochromatin recombination rates reveals consistent negative correlations between recombination rate and genome size, no matter which ploidy level was selected. For the 100 permutations of genome size (pg) for species with multiple ploidy levels, the metacorrelation between recombination rate (cM/pg) and genome size in pg has mean *r* = −0.70 with a 95 % confidence interval (−0.683, −0.721) and *p* < 0.001. The metacorrelation of recombination rate (cM/pg) and genome size in Mb has mean *r* = −0.35 with a 95 % confidence interval (−0.31, −0.38) and *p* < 0.001. This suggests that genome size is negatively correlated with recombination rate, and this result is robust to both different ploidy levels between and within species as well as error in genome size estimates from assemblies.

### Recombination Rate and dN/dS

We were interested if purifying selection is associated with increasing recombination rate more frequently than positive selection, which is observed within populations of model organisms such as *Drosophila melanogaster* [38]. Low *dN/dS* values can be interpreted as evidence for purifying selection. Therefore, if recombination rate is also associated with purifying selection at a macroevolutionary scale, we should find more evidence for negative correlations between recombination rate and *dN/dS* than positive correlations across genes.

**Fig. 3:**
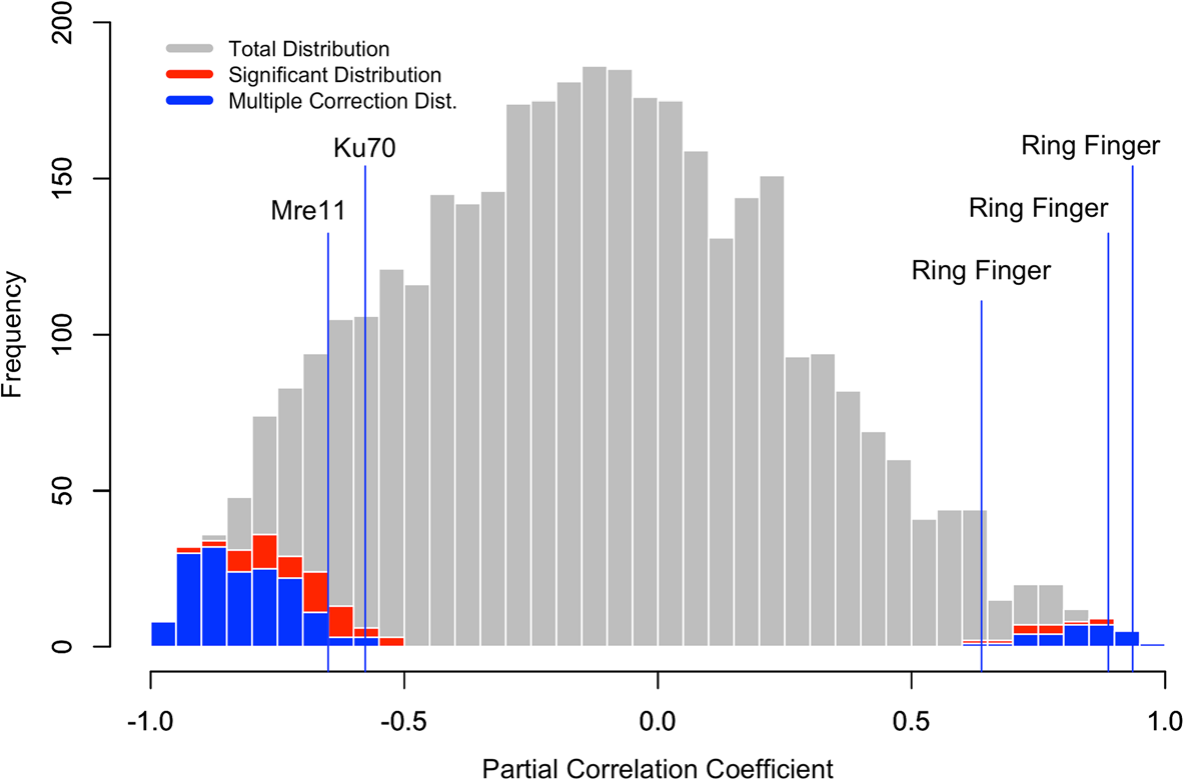
Distributions of correlation coefficients from COEVOL. Only results from chains that converged are displayed. A negative correlation coefficient indicates a relationship between global recombination rate and purifying selection for a gene while a positive correlation indicates a relationship between recombination rate and relaxed selection for a gene. The total distribution is displayed in grey, while the significant distribution for posterior probabilities of 0.025 and 0.975 are shown in red, and corrected posterior probabilities for 5 % significant results are in blue

Recombination rate is correlated with *dN/dS* for 6.9 % of 3748 genes using nominal two-tailed posterior probability cutoffs of 0.025 and 0.975. Since we are using posterior probabilities, it is inappropriate to correct for the family-wise error rate or the false discovery rate by treating them as *p*-values. Therefore, we correct our nominal posterior probability cutoffs to achieve desired 5 % significant results. Of the significant results, 16 % of these are positive correlations (posterior probability > 0.983), while 84 % are negative correlations (posterior probability < 0.017) (Fig. 3). This indicates that most genes experience more effective purifying selection as global recombination rate increases, while few genes experience higher *dN/dS*, which could be due to relaxed selective pressures or to increased efficacy of positive selection with increasing recombination rate [88]. Hidden paralogy is always a concern when investigating plant nuclear genes, but this likely would only make convergence more difficult, generating uncertainty and lowering ESS at duplication nodes. Overall, increasing recombination rate is associated with stronger purifying selection for both the significant pool and non-significant pool of corrected tests (*Χ*^2^ = 262.44, *p* < 0.001). These results imply that the rate of recombination, a population genetic process, can influence the patterns of molecular evolution across species.

We also found evidence of correlations between recombination rate and the *dN/dS* of several genes linked to recombination. Ring finger domains play a critical role as ubiquitin ligases [89], and it is thought that ring finger domain containing proteins assist with initiating double stranded breaks [90]. Ring finger domain containing proteins may take part in the meiotic recombination mechanism in plants, since they are associated with early protein-protein interactions for crossover formation in model systems like yeast and *Caenorhabditis elegans* [91–93]. Sequence variation in the ring finger domain containing protein RNF212 also is correlated with recombination rate in humans [94]. Three ring fingers are significantly positively correlated with recombination rate in this study (Fig. 3), but there is no overall enrichment of zinc fingers in the COEVOL results (Fisher exact test, *p* = 0.515; Additional file 1: Table S7). Likewise, the well characterized Mre11, which participates in heteroduplex resolution and possibly telomere maintenance, and the nonhomologous end joining protein Ku70 both experience stronger purifying selection (i.e., lower *dN/dS*) as recombination increases (Fig. 3), but meiotic recombination proteins are not enriched in the COEVOL results either (Fisher exact test, *p* = 0.071; Additional file 1: Table S8).

### Composition Biases across Genes Associated with Recombination Rate

**Fig. 4:**
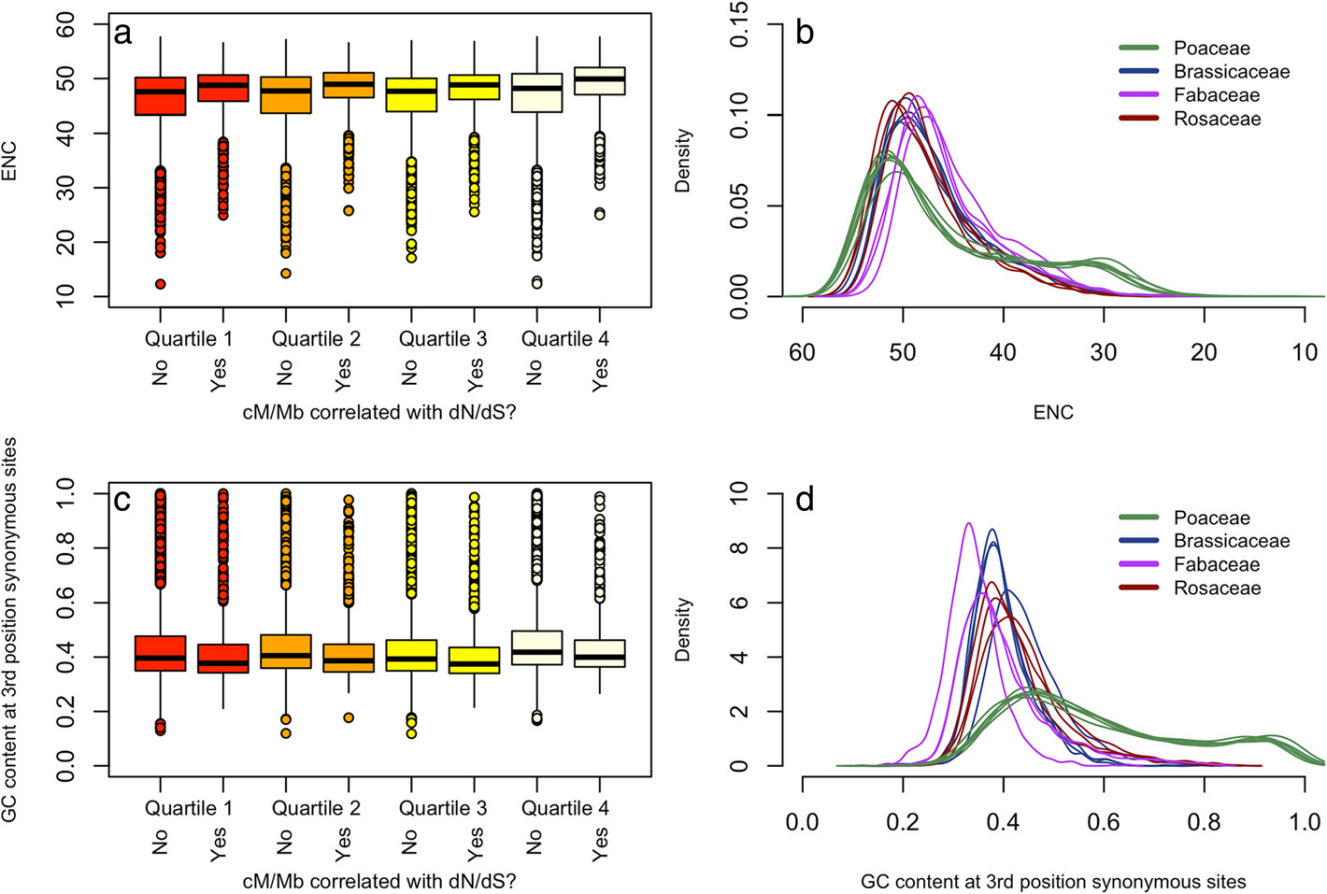
**a** Distributions of ENC binned by species with the bottom, second, third, and top quartiles of global recombination rate, pooled from not significant and significant genes from the 3748 alignments used for COEVOL **b** The total distributions of ENC for the 3748 COEVOL genes for species from the best represented plant families in this study. **c** Distributions of 3GC^S^ ordered by recombination rate. **d** Complementary distribution of 3GC^S^ shows similar patterns of codon bias and GC bias at 3^rd^ position 4-fold degenerate sites in Poaceae vs other plant families best represented in this study

Finally, we tested for associations between recombination rate and nucleotide compositional biases, in an attempt to understand the degree that GC biased gene conversion might influence covariation between recombination rate and *dN/dS*. Recombination rate was not correlated with the genome average ENC or 3GC^S^ (Additional file 3: Figure S2). However, recombination rate could still affect sequence variation in genes in which *dN/dS* is correlated with global recombination rate. We used the alignments analyzed with COEVOL to examine the distribution of ENC and 3GC^S^ for genes in which the *dN/dS* is not associated with recombination rate and the pool of genes in which *dN/dS* is associated with recombination rate (Additional file 1: Table S9–S12). For simplicity, we binned distributions into recombination rate quartiles (Figs. 4a and 4c). We do not observe a pattern, suggesting that genes in which *dN/dS* is correlated with global recombination rate have a stronger bias in codon usage or 3GC^S^ than genes in which *dN/dS* is not correlated with global recombination rate. Detectable composition biases in our data appear to be lineage specific, such as strong biases in both ENC and 3GC^S^ in grasses (Figs. 4b and 4d).

## Discussion

Global recombination rate appears to be evolutionarily labile across angiosperms (Fig. 1; Additional file 1: Table S4), and even relatively closely related congeners can have very different recombination rates (e.g., *Solanum lycopersicum* and *S. tuberosum*). Jaramillo-Correa et al. [95] found evidence of phylogenetic structure in recombination rate estimates (cM/Mb) across 81 seed plant species. However, Jaramillo-Correa et al. [95] included conifers, which have low recombination rates and likely contributed heavily to the observed phylogenetic structure. Still, this study indicates that global recombination rate is strongly associated with the evolution of genome structure and patterns of molecular evolution in angiosperms (Figs. 2 and 3).

We find a strong negative correlation between global recombination rate and genome size as well as the total proportion of LTR retrotransposons. This result is consistent with many previous studies and expectations (Fig. 2; [5, 14, 96, 97]). In contrast, Ross-Ibarra [10] observed that recombination rate increases with genome size in angiosperms, when measuring global recombination rate as the number of chiasmata per chromosome arm. It is unclear what mechanism would produce a positive correlation between recombination rate and genome size, but still, this does not necessarily contradict our results, as the recombination rate estimates are not directly comparable. While there may be some error in our estimates of recombination rate, linkage map lengths were averaged across multiple populations for each species, and the correlations were similar whether we used the scaffold assembly size or C-values to represent genome size (Additional file 1: Table S5 and Table S6). Furthermore, any error in recombination rate estimates should not strengthen the correlation with genome size. The negative correlation between recombination rate and genome size also makes sense mechanistically. LTR retrotransposons, which play a large role in expanding the genome size of plants, may generally have deleterious effects in gene rich euchromatin, and meiotic recombination can facilitate removal of LTR retrotransposons by unequal or intra-strand crossing over [14]. The relationship between recombination rate and LTR retrotransposon content may entirely explain the relationship between recombination rate and genome size, as recombination rate is not negatively correlated with genome size after removing LTR retrotransposon content (Fig. 2a). However, many correlates of global recombination rate are also likely correlates of genome size, and therefore, our analyses do not prove a causal relationship between global recombination rates and genome size.

We found no evidence linking recombination rate and 3GC^S^ or ENC globally (Figure S2) or across angiosperm genes where recombination rate is correlated with *dN/dS* (Fig. 4d and 4b)*.* However, grasses have strong composition biases compared to the non-monocots represented in this study (Fig. 4). GC biased gene conversion has been observed in grasses [32, 98, 99], which may indicate that some effects of recombination are specific to certain clades but not pervasive across all angiosperms. For example, GC biased gene conversion appears to be reduced in self-fertilizing species [30, 100, 101]. Thus, there may be a combination of biological factors necessary for GC biased gene conversion to occur.

Recombination is generally restricted to gene rich regions of the genome [60], and therefore, it is sensible to correct for the nonrandom distribution of crossovers when calculating recombination rates (e.g., [10]). There was a positive correlation between global and euchromatin corrected recombination rates, and correlation coefficients estimated using the euchromatin corrected recombination rate were generally weaker, but consistent with those estimated using the global recombination rate. One exception is that euchromatin corrected recombination rate was negatively correlated with genome compactness (Figure S1), which may better characterize the euchromatic portion of the genome.

Interestingly, the euchromatin corrected recombination rate is strongly positively associated with the average number of genes in a gene family, while global recombination rate is not. Cook’s distance applied to a linear regression model indicates that the relationship between euchromatic recombination rate and average gene family size is largely influenced by contrasts between *Solanum tuberosum* and *S.* lycopersicum, *Manihot esculenta* and *Populus trichocarpa*, *Zea mays* and *Sorghum bicolor*, and the ancestral state of Asterids (represented only by *S. tuberosum* and *S. lycopersicum* in the euchromatin corrected data) and Rosids. We applied Kendall’s tau to the data because a nonparametric test should be less sensitive to possible outliers, and a significant positive association remains (*τ* = 0.4118, *p* = 0.017). Whole genome duplication alone also cannot explain these contrasts. For example, *S. bicolor* has a larger average gene family size than *Z. mays*, despite *Z. mays* having undergone a lineage specific whole genome duplication since its divergence from *S. bicolor* [102]. Additionally, *S. tuberosum* and *S. lycopersicum* share a whole genome triplication [103], yet the genome of *S. lycopersicum* is composed of much more heterochromatin and has a larger average gene family size. The positive correlation between the euchromatin corrected recombination rate and average gene family size also persists even if the largest gene families are removed (Additional file 1: Table S13). Thus, this association is not due to massive expansion of a few families or clustering errors

The positive association between euchromatin corrected recombination rate and gene family size may be due to a link between recombination and tandem duplication rate, since more duplicate genes are located near sites of recombination [46–48]. Conversely, gene loss rates also may be lower near regions of high recombination due to the presence of strong purifying selection. Recombination rate can affect the time to fixation and the efficacy of selection for duplicate genes [49, 104]. Specifically, the probability of subfunctionalization or neofunctionalization of a newly duplicated gene and the preservation of that gene is maximized under free recombination [49–51]. Lower levels of recombination will ultimately reduce the probability of preservation of any given duplicate, assuming the duplication event itself is not selected for due to additive dosage effects [51]. Variation in angiosperm gene content is often discussed in the context of whole genome duplications [105–107], but our results suggest that recombination, independent of whole genome duplications, may be critical for creating and maintaining gene copy variation.

Recombination is linked to efficacy of purifying selection in populations [38] and also within genomes. Generally, genes in regions of the genome with high recombination rates should have lower *dN/dS* than genes in regions of low recombination. Campos et al. [38] found that regions with crossovers have seven times the synonymous nucleotide diversity of regions without crossovers in a population of *Drosophila melanogaster*, which corresponds with findings in populations of *Arabidopsis lyrata* [108], and patterns of SNP variation in humans [109]. Our analyses suggest that the increased efficacy of selection due to recombination is also observable on a macroevolutionary scale across angiosperms. Our analyses linking lower *dN/dS* with increased recombination rates in many genes support the hypothesis that purifying selection acts more effectively in species with higher global recombination rates (Fig. 3). Although we find a small proportion of genes where *dN/dS* increases with global recombination rate, it is not certain that this is the result of hitchhiking. However, patterns of *dN/dS* variation across genes imply a role for background selection in plant genome evolution. While several genes involved with meiotic recombination were analyzed in this study, these follow the broader pattern of correlations between recombination rate and genes. These results suggest that while a small proportion of recombination associated genes are correlated with recombination rate, there is not likely any selective pressures acting on these genes as a group to modify recombination rate.

While our results suggest a role for recombination in shaping macroevolutionary patterns of genome architecture and molecular evolution in plants, well-known covariates of recombination rate, genome size, and substitution rates, such as effective population size [110], could strongly affect the results. Obtaining estimates of effective population size can be challenging [111], and we could not incorporate effective population size into this study due to the limited availability of these estimates. Regardless, our results suggest that Hill-Robertson effects may have macroevolutionary consequences on both the interspecific rates of molecular evolution and the average size of gene families among species. Other gene-specific factors, such as gene function, tissue specificity, expression level, and architectural features of the genes, may further elucidate the possible relationship between interspecific recombination rate evolution and patterns of variation in *dN/dS*, as they have for intraspecific studies (e.g., [112–114]), since these also covary with rates of molecular evolution and recombination rate [115, 116]. Associations between recombination rate and selection are typically weaker in plants than other eukaryotes [117, 118], so codon models that allow for among site rate heterogeneity in *dN/dS* on branches might also help further reveal the relationship between local recombination rate and *dN/dS* across species [119].

## Conclusions

Although genomic data has enabled many insights into plant evolution, the role of population level evolutionary processes on macroevolutionary patterns is still largely unknown. Understanding the impacts of recombination rate variation, in addition to effective population size, selection, and mutation, is necessary for elucidating genome evolution. The results presented in this study are largely consistent with previous intraspecific studies [17–20, 29, 30]. While it is unclear if our results reflect the role of recombination in genome evolution or a covariate of recombination rate, taken together with previous research, they suggest recombination rate affects genome architecture and the distribution of *dN/dS* across angiosperm species. The effects include removal of LTR retrotransposons and influencing gene duplication and loss. Recombination rate variation may not only explain the rate at which tandem duplicates arise, but also the preservation of duplicate genes through increased efficacy of purifying selection.

### Availability of Supporting Data

The data set supporting the results of this article are available through the Dryad digital repository, doi:10.5061/dryad.10nh8. Additional scripts for performing COEVOL analyses, calculating ENC, and calculating 3GC^S^ are available on GPT’s github page (https://github.com/gtiley/ResearchSupplements/tree/master/Tiley_and_Burleigh_2015-Recombination). Tools for making codon alignments are available at https://github.com/gtiley/Alignment_Tools/tree/master/Codon_Alignment.

## Additional files

Additional file 1: Table S1. Citations for the sequenced genome examined in this study, pachytene chromosome smears used for differentiating euchromatin and heterochromatin, and genetic maps used to estimate recombination rate are provided. **Table S2.** Map lengths, number of markers for genetic maps, corrected map lengths and total recombination rates used in study. **Table S3.** Genome sizes in pg for all available ploidy levels within a species, taken from the Kew C-Value database. **Table S4.** Blomberg’s K test statistics for traits examined in this study. **Table S5.** correlation coeffiecients for resampled data sets of cM/C-value vs C-value used for meta correlation. **Table S6.** Correlation coeffiecients for resampled data sets of cM/C-value vs Mb used for meta correlation. **Table S7.** List of genes analyzed with coevol annotated as zinc fingers. **Table S8.** List of genes analyzed with coevol with know recombination functions. **Table S9.** Distribution of ENC for significant coevol genes. Rows do not correspond to the same alignment, but columns represent vectors of ENC for all alignments for each species. **Table S10.** Distribution of ENC for NOT significant coevol genes. rows do not correspond to the same alignment, but columns represent vectors of ENC for all alignments for each species. **Table S11.** Distribution of 3GCS for significant coevol genes. rows do not correspond to the same alignment, but columns represent vectors of 3GCS for all alignments for each species. **Table S12.** Distribution of 3GCS for NOT significant coevol genes. **Table S13.** Average gene family sizes for species in study based on 11,250 clusters that span the root of the species tree in **Fig. 1.** (PDF 3384 kb) Additional file 2:
**Supplemental Literature.** (PDF 136 kb) Additional file 3: Figure S1. Independent contrast plots of recombination rate vs genome compactness and chromosome number. Lines on each plot are the best-fit linear regression. The Pearson’scorrelation coefficient (*r*) and *p*-value (*p*) are given on each plot. **Figure S2.** Independent contrast plots of recombination rate vs GC content at 3rd position synonymous sites (3GCS) and codon bias (ENC). Lines on each plot are the best-fit linear regression. The Pearson’s correlation coefficient (*r*) and p-value (*p*) are given on each plot. (PDF 194 kb)
