# Erratum to: The relationship of recombination rate, genome structure, and patterns of molecular evolution across angiosperms

**DOI:** 10.1186/s12862-015-0525-8

**Published:** 2015-11-09

**Authors:** George P. Tiley, J. Gordon Burleigh

**Affiliations:** Department of Biology, University of Florida, Bartram-Carr Hall, Gainesville, 32611 FL USA

## Erratum

The original version of this article [1] unfortunately contained a mistake. The author list was presented with a mistake, Dr J. Gordon Burleigh was incorrectly listed as “Gordon Burleigh” in both the PDF and HTML versions of this manuscript. The corrected author list is provided below:

George P. Tiley* and J. Gordon Burleigh

The original article [1] was updated to reflect this change.
