# Supplementary material for: The relationship of recombination rate, genome structure, and patterns of molecular evolution across angiosperms
Source: BMC Evol Biol. 2015 Sep 16;15:194. doi: 10.1186/s12862-015-0473-3 (PMC4574184; doi:10.1186/s12862-015-0473-3)
Supplement: Additional file 2: — Supplemental Literature. (PDF 136 kb) [file 12862_2015_473_MOESM2_ESM.pdf]

Agren JA, Wang W, Koenig D, Neuffer, Weigel D, Wright SI. 2014. Mating system shifts and transposable element evolution in the plant genus *Capsella*. *BMC Genomics* 15:602

Akkaya MS, Shoemaker RC, Specht JE, Bhagwat AA, Cregan PB. 1995. Integration of simple sequence repeat DNA markers into a soybean linkage map. *Crop Sci.* 35: 1439-1445

Anderson LK, et al. 2003. High-resolution crossover maps for each bivalent of *Zea mays* using recombination nodules. *Genetics* 165:849-865

Anderson LK, Lai A, Stack SM, Rizzon C, Gaut BS. 2006. Uneven distribution of expressed sequence tag loci on maize pachytene chromosomes. *Genome Res.* 16:115-122

Arabidopsis Genome Initiative. 2000. Analysis of the genome sequence of the flowering plant *Arabidopsis thaliana*. *Nature* 408:796–815

Ashrafi H, Kinkade M, Fooland MR. 2009. A new genetic linkage map of tomato based on a *Solanum lycopersicum* x *S. pimppinelliflium* RIL population displaying locations of candidate pathogen response genes. *Genome* 52:935-956

Bennetzen JL, et al. 2012. Reference genome sequence of the model plant *Setaria*. *Nat. Biotechnol.* 30(6):555-561

Berlin S, Lagercrantz U, von Arnold S, öst T, Rönnberg-Wästljung AC. 2010. High-density linkage mapping and evolution of paralogs and orthologs in *Salix* and *Populus*. *BMC Genomics* 11:129

Bernet GP, Fernandez-Ribacoba JF, Carbonell EA, Asins MJ. 2010. Comparative genome-wide segregation analysis and map construction using a reciprocal cross design to facilitate citrus germplasm utilization. *Mol. Breeding* 25:659-673

Berr A, et al. 2006. Chromosome arrangement and nuclear architecture but not centromeric sequences are conserved between *Arabidopsis thaliana* and *Arabidopsis lyrata*. *The Plant Journal* 48:771-783

Bhattramakki D, Dong J, Chhabra AK, Hart GE. 2000. An integrated SSR and RFLP linkage map of *Sorghum bicolor* (L.) Moench. *Genome* 43:988-1002

Blair MW, et al. 2003. Development of a genome-wide anchored microsatellite map for common bean (*Phaseolus vulgaris* L.). *Theor. Appl. Genet.* 107:1362-1374

Boivin K, Acarkan A, Mbulu R, Clarenz O, Schmidt R. 2004. The Arabidopsis genome sequence as a tool for genome analysis in Brassicaceae. A comparison of the Arabidopsis and *Capsella rubella* genomes. *Plant Physiology* 135:735-744

Bradshaw HD, et al. 1994. Molecular genetics of growth and development in *Populus*. III. A genetic linkage map of a hybrid poplar composed of RFLP, STS, and RAPD markers. *Theor. Appl. Genet.* 89:167-178

Brubaker CL, Paterson AH, Wendel JF. 1999. Comparative genetic mapping of allotetraploid cotton and its diploid progenitors. *Genome* 42:184-203

Celton J, Tustin DS, Chagné D, Gardiner SE. 2009. Construction of a dense genetic linkage map for apple rootstocks using SSRs developed from *Malus* ESTs and *Pyrus* genomic sequences. *Tree Genetics and Genomes* 5:93-107

Cervera M, et al. 2001. Dense genetic linkage maps of three Populus species (*Populus deltoides, P. nigra,* and *P. trichocarpa*) based on AFLP and microsatellite markers. *Genetics* 158:787-809

Chang C, Bowman JL, DeJohn AW, Lander ES, Meyerowitz EM. 1988. Restriction fragment length polymorphism map for *Arabidopsis thaliana*. *Proc. Natl. Acad. Sci. USA* 85:6856-6860

Chen X, Temnykh S, Xu Y, Cho YG, McCouch SR. 1997. Development of a microsatellite framework map providing genome-wide coverage in rice (*Oryza sativa* L.). *Theor. Appl. Genet.* 95:553-567

Chen C, al. 2007. Construction of a sequence-tagged high-density genetic map of papaya for comparative structural and evolutionary genomics in Brassicales. *Genetics* 177:2481-2491

Chen C, et al. 2008. EST-SSR genetic maps for *Citrus sinensis* and *Poncirus trifoliata*. *Tree Genetics and Genomes* 4:1-10

Cheng Z, Buell CR, Wing RA, Gu M, Jiang J. 2001. Toward a cytological characterization of the rice genome. *Genome Res.* 11:2133-2141

Cho YG, et al. 1998. Integrated map of AFLP, SSLP and RFLP markers using a recombinant inbred population of rice (*Oryza sativa* L.) *Theor Appl. Genet.* 97:370-380

Choi H, et al. 2004. A sequence-based genetic map of *Medicago truncatula* and comparison of marker colinearity with *M. sativa*. *Genetics* 166:1463-1502

Choi SR, et al. 2007. The reference genetic linkage map for the multinational *Brassica rapa* genome sequencing project. *Theor. Appl. Genet.* 115:777-792

Chyi Y, Hoenecke ME, Sernyk JL. 1992. A genetic linkage map of restriction fragment length polymorphism loci for *Brassica rapa* (syn. *campestris*). *Genome* 35:746-757

Cloutier S, Ragupathy R, Niu Z, Duguid S. 2011. SSR-based linkage map of flax (*Linum usitatissimum* L.) and mapping of QTLs underlying fatty acid composition traits. *Mol. Breeding* 28:437-451

Connor PJ, Brown SK, Weeden NF. 1997. Randomly amplified polymorphic DNA-based genetic linkage maps of three apple cultivars. *J. Amer. Soc. Hort. Sci.* 122(3):350-359

Cregan PB, et al.1999. An integrated genetic linkage map of the soybean genome. *Crop Sci.* 39:1464-1490

Crouzillat D, et al. 1996. *Theobroma cacao* L.: a genetic linkage map and quantitative trait loci analysis. *Theor. Appl. Genet.* 93:205-214

Davis TM, Yu H. 1997. A linkage map of the Diploid Strawberry, *Fragaria vesca*. *Journal of Heredity* 88:215-221

Dettori MT, Quarta R, Verde I. 2001. A peach linkage map integrating RFLPs, SSRs, RAPDs, and morphological markers. *Genome* 44:783-790

Dirlewanger E, Pronier V, Parvery C, Rothan C, Guye A, Monet R. 1998. Genetic linkage map of peach [*Prunus persica* (L.) Batsch] using morphological and molecular markers. *Theor. Appl. Genet.* 97:888-895

Dirlewanger E, et al.2006. Development of a second-generation genetic linkage map for peach [*Prunus persica* (L.) Batsch] and characterization of morphological traits affecting flower and fruit. *Tree Genetics and Genomes* 3:1-13

Eucalyptus grandis Genome Project 2010, http:://www.phytozome.net/eucalyptus

Fazio G, Staub JE, Stevens MR. 2003. Genetic mapping and QTL analysis of horticultural traits in cucumber (*Cucumis sativus* L.) using recombinant inbred lines *Theor. Appl. Genet.* 107:864-874

Fishman L, Kelly AJ, Morgan E, Willis JH. 2001. A genetic map in the *Mimulus guttatus* species complex reveals transmission ratio distortion due to heterospecific interactions. *Genetics* 159:1701-1716

Fransz P, et al. 1998. Cytogenetics for the model system *Arabidopsis thaliana*. *The Plant Journal* 13(6):867-876

Freyre R, et al. 1998. Towards an integrated linkage map of common bean. 4. Development of a core linkage map and alignment of RFLP maps. *Theor. Appl. Genet.* 97:847-856

Ganal MW, et al. 2011. A large maize (*Zea mays* L.) SNP genotyping array: Development and germplasm genotyping, and genetic mapping to compare with the B73 reference genome. *PLoS ONE* 6(12):e28334

Garvin DF, et al. 2010. An SSR-based genetic linkage map of the model grass *Brachypodium distachyon*. *Genome* 53:1-13

Gebhardt C, et al. 1989. RFLP analysis and linkage mapping in *Solanum tubersum*. *Theor. Appl. Genet.* 78:65-75

Grando MS, et al. 2003. Molecular linkage maps of *Vitis vinifera* L. and *Vitis riparia* Mchx. *Theor. Appl. Genet.* 106:1213-1224

Grattapaglia D, Sederoff R. 1994. Genetic linkage maps of *Eucalyptus grandis* and *Eucalyptus urophylla* using a pseudo-testcross: Mapping strategy and RAPD markers. *Genetics* 137:1121-1137

Haploid Clementine Genome, International Citrus Genome Consortium, 2011, http://int-citrusgenomics.org/, http:://www.phytozome.net/Clementine

Harushima Y, et al. 1998. A high-density rice genetic linkage map with 2275 markers using a single F_2 population. *Genetics* 148:479-494

Hellsten U, et al. 2013. Fine-scale variation in meiotic recombination in *Mimulus* inferred from population shotgun sequencing. *PNAS*110:19478-19482

Huo N, et al. 2011. Comparison of a high-density genetic linkage map to genome features in the model grass *Brachypodium distachyon*. *Theor. Appl. Genet.* 123:455-464

Hu TT, et al. 2011. The *Arabidopsis lyrata* genome sequence and the basis of rapid genome size change. *Nat. Genet.* 43(5):476-81

Huang S, et al. 2009. The genome of the cucumber, *Cucumis sativus* L. *Nat. Genet.* 41(12):1275-1281

International *Brachypodium* Initiative. 2010. Genome sequencing and analysis of the model grass *Brachypodium distachyon*. *Nature* 463(7282):763-8

International Rice Genome Sequencing Project. 2005. The map-based sequence of the rice genome. *Nature* 436:793-800

Jacobs JME, et al. 1995. A genetic map of potato (*Solanum tuberosum*) integrating molecular markers, including transposons, and classical markers. *Theor. Appl. Genet.* 91:289-300

Jaillon O, et al. 2007. The grapevine genome suggests ancestral hexaploidization in major angiosperm phyla. *Nature* 449(7161):463-467

Jelenkovic G, Harrington E. 1972. Morphology of the pachytene chromosomes in *Prunus persica*. *Can. J. Genet. Cytol.* 14:317-324

Jia X, et al. 2009. Development and genetic mapping of SSR markers in foxtail millet [*Setaria italica* (L.) P. Beauv.]. *Theor. Appl. Genet.* 118:821-829

Kennard WC, et al. 1994. Linkages among RFLP, RAPD, isozyme, disease-resistance, and morphological markers in narrow and wide crosses of cucumber. *Theor. Appl. Genet.* 89:42-48

Kim JS, et al. 2006. A sequence-tagged linkage map of *Brassica rapa*. *Genetics* 174:29-39

Kim JS, et al. 2005. Comprehensive molecular cytogenetic analysis of Sorghum genome architecture: distribution of euchromatin, heterochromatin, genes and recombination in comparison to rice. *Genetics* 171:1963-1976

Kole C, Kole P, Vogelzang R, Osborn TC. 1997. Genetic linkage map of a *Brassica rapa* recombinant inbred population. *The Journal of Heredity* 88(6):553-557

Koo D, Choi H, Cho J, Hur Y, Bang J. 2005. A high-resolution karyotype of cucumber (*Cucumis sativus* L. ‘Winter Long’) revealed by C-banding, pachytene analysis, and RAPD-aided fluorescence in situ hybridization. *Genome* 48:534-540

Koo D, Plaha P, Lim TP, Hur Y, Bang J, 2004. A high-resolution karyotype of *Brassica rapa* ssp. *pekinensis* revealed by pachytene analysis and multicolor fluorescence in situ hybridization. *Theor. Appl. Genet.* 109:1346-1352

Kuittinen H, et al. 2004. Comparing the linkage maps of the close relatives *Arabidopsis lyrata* and *A. thaliana*. *Genetics* 168:1575-1584

Kulikova O, et al. 2001. Integration of the FISH pachytene and genetic maps of *Medicago truncatula*. *The Plant Journal* 27(1):49-58

Kunkeaw S, Tanghatsornruang S, Smith DR, Triwitayakorn K. 2010. Genetic linkage map of cassava (*Manihot esculenta* Crantz) based on AFLP and SSR markers. *Plant Breeding* 129:112-115

Lanaud C, et al. 1995. A genetic linkage map of *Theobroma cacao* L. *Theor. Appl. Genet.* 91:987-993

Lark KG, et al. 1993. A genetic map of soybean (*Glycine max* L.) using an intraspecific cross of two cultivars: 'Minosy' and 'Noir 1'. *Theor. Appl. Genet.* 86:901-906

Liebhard R, Koller B, Gianfranceschi L, Gessler C. 2003. Creating a saturated reference map for the apple (*Malus x domestica* Borkh.) genome. *Theor. Appl. Genet.* 106:1497-1508

Lin J, Ritland K. 1996. Construction of a genetic linkage map in the wild plant *Mimulus* using RAPD and isozyme markers. *Genome* 39:63-70

Lodhi MA, Daly MJ, Ye G, Weeden NF, Reisch BI. 1995. A molecular marker based linkage map of *Vitis*. *Genome* 38:786-794

Magoon ML, Krishnan R, Bai KV. 1969. Morphology of the pachytene chromosomes and meiosis in *Manihot esculenta* Crantz. *Cytolgia* 34:612-626

Maliepaard C, et al. 1998. Aligning male and female linkage maps of apple (*Malus pumila* Mill.) using multi-allelic markers. *Theor. Appl. Genet.* 97:60-73

Matsumoto E, Yasui C, Ohi M, Tsukada M. 1998. Linkage analysis of RFLP markers for clubfoot resistence and pigmentation in Chinese cabbage (*Brassica rapa* spp. *pekinesis*). *Euphytica* 104:79-86

Menz MA, et al. 2002. A high-density genetic map of *Sorghum bicolor* (L.) Moench based on 2926 AFLP, RFLP, and SSR markers. *Plant Molecular Biology* 48:483-499

Miao H, et al. 2011. A linkage map of cultivated cucumber (*Cucumis sativus* L.) with 248 microsatellite marker loci and seven genes for horticulturally important traits. *Euphytica* 182:167-176.

Mimulus Genome Project, DoE Joint Genome Institute

Ming R, et al. 2008. The draft genome of the transgenic tropical fruit tree papaya (*Carica papaya* Linnaeus). *Nature* 452(7190):991-996

Miranda M, Ikeda F, Endo T, Moriguchi T, Omura M. 1997. Comparative analysis of heterochromatin in *Citus*, *Poncirus*, and *Fortunella* chromosomes. *Chromosome Research* 5:86-92

Moscone EA, Klein F, Lambrou M, Fuchs J, Schweizer D. Quantitative karyotyping and dual-color FISH mapping of 5S and 18S-25S rDNA probes in the cultivated *Phaseolus* species (Leguminosae). 42:1224-1233

Motomayor JC, et al. 2013. The genome sequence of the most widely cultivated cacao type and its use to identify candidate genes regulating pod color. *Genome Biology* 14:R53

Mun J, et al. 2006. Distribution of microsatellites in the genome of *Medicago truncatula*: A resource of genetic markers that integrate genetic and physical maps. *Genetics* 172:2541-2555

Myburg AA, Grattapaglia D, Tuskan GA, Hellsten U, Hayes RD, Grimwood J, Jenkins J, Lindquist E, Tice H, Bauer D, et al. 2014. The genome of *Eucalyptus grandis*. *Nature* 510:356-362

Myburg AA, Griffin AR, Sederoff RR, Whetten RW. 2003. Comparative genetic linkage maps of *Eucalyptus grandis, Eucalyptus globulus* and their F_1 hybrid based on a double pseudo-backcross mapping approach. *Theor. Appl. Genet.* 107:1028-1042.

Nam H, et al. 1989. Restriction fragment length polymorphism map of *Arabidopsis thaliana*. *The Plant Cell* 1:699-705.

Oh TJ, Gorman M, Cullis CA. 2000. RFLP and RAPD mapping in flax (*Linum usitatissimum*). *Theor. Appl. Genet.* 101:590-593

Okada M, et al. 2010. Complete switchgrass genetic maps reveal subgenome collinearity, preferential pairing and multilocus interactions. *Genetics* 185:745-760

Oliveira RP, Cristofani M, Machado MA. 2004. Genetic linakage maps of ‘Pêra’ sweet orange and ‘Cravo’ mandarin with RAPD markers. *Pesq. agropec. bras., Brasilia* 39(2):159-165

Ollitrault P, et al. 2012. A reference genetic map of *C. clementina* hort. Tan.; citrus evolution inferences from comparative mapping. *BMC Genomics* 13:593

Ouyang S, et al. 2007. The TIGR rice genome annotation resource: improvements and new features. *Nuc. Acids Res.* 35:D833-7

Panicum virgatum v1.1, DOE-JGI, http:://www.phytozome.net/panicumvirgatum

Paterson AH, et al. 2009. The *Sorghum biocolor* genome and the diversification of grasses. *Nature* 457(7229)551-556

Paterson AH, et al. 2012. Repeated polyploidization of *Gossypium* genomes and the evolution of spinnable cotton fibers. *Nature* 492:423-428

Peng Y, Schertz KF, Cartinhour S, Hart GE. 1999. Comparative genome mapping of *Sorghum bicolor* (L.) Moench using an RFLP map contructed in a population of recombinant inbred lines. *Plant Breeding* 118:225-235

Pereira MG, et al. 1994. Construction of an RFLP map in sorghum and comparative mapping in maize. *Genome* 37:236-243

Peterson DG, Price HJ, Johnston JS, Stack SM. 1996. DNA content of heterochromatin and euchromatin in tomato (*Lycopersicon esculentum*) pachytene chromosomes. *Genome* 39:77-82

Price AH, Steele KA, Moore BJ, Barraclough PB, Clark LJ. 2000. A combined RFLP and AFLP linkage map of upland rice (*Oryza sativa* L.) used to identify QTLs for root-penetration ability. *Theor. Appl. Genet.* 100:49-56

Prochnik S, et al. 2012. The cassava genome: current progress, future directions. *Trop. Plant Biol.* 5(1):88-94

Pugh T, et al. 2004. A new cacao linkage map based on codominant markers: development and integration of 201 new microsatellite markers. *Theor. Appl. Genet.* 108:1151-1161

Rabbi IY, Kulembeka HP, Masumba E, Marri PR, Ferguson M. 2012. An EST-derived SNP and SSR genetic linkage map of cassava (*Manihot esculenta* Crantz). *Theor. Appl. Genet.* 125:329-342

Reiter RS, et al.1992. Global and local genome mapping in *Arabidopsis thaliana* by using recombinant inbred lines and random amplified polymorphic DNAs. *Proc. Natl. Acad. Sci. USA* 89:1477-1481

Ren Y, et al. 2009. An integrated genetic and cytogenetic map of the cucumber genome. *PLoS ONE* 4(6):e5795

Riaz S, Dangl GS, Edwards KJ, Meredith CP. 2004. A microsatellite marker based framework l inkage map of *Vitis vinifera* L. *Theor. Appl. Genet.* 108:864-872

Risterucci AM, et al. 2000. A high-density linkage map of *Theobroma cacao* L. *Theor. Appl. Genet.* 101:948-955

Rodríguez-Suárez C, Méndez-Vigo B, Pañeda A, Ferreira JJ, Giraldez R. 2007. A genetic linkage map of *Phaseolus vulgaris* L. and localization of genes for specific resistance to six races of anthracnose (*Colletotrichum lindemuthianum*). *Theor. Appl. Genet.* 114:713-722

Rong J, et al. 2004. A 3347-locus genetic recombination map of sequence-tagged sites reveals features of genome organization, transmission, and evolution of cotton (*Gossypium*). *Genetics* 166:389-417

Salmaso M, et al. 2008. A grapevine (*Vitis vinifera* L.) genetic map integrating the position of 139 expressed genes. *Theor. Appl. Genet.* 116:1129-1143

Sargent DJ, et al. 2004. A genetic linkage map of microsatellite, gene-specific, and morphological markers in diploid *Fragaria*. *Theor. Appl. Genet.* 109:1385-1391

Sargent DJ, et al. 2006. An enhanced microsatellite map of diploid *Fragaria*. *Theor. Appl. Genet.* 112:1349-1359

Schmutz J, et al..2010. Genome sequence of palaeopolyploid soybean. *Nature* 463(7278):178-183

Schmutz J, et al. 2014. A reference genome for common bean and genome-wide analysis of dual domestications. *Nature Genetics* 46:707-713

Schnable PS, et al. 2009. The B73 maize genome: complexity, diversity, and dynamics. *Science* 326(5956):1112-1115

Shirasawa K, et al. 2010. SNP discovery and linkage map construction in cultivated tomato. *DNA Research* 17:381-391

Shirasawa K, et al. 2010. An interspecific linkage map of SSR and intonic polymorphism markers in tomato. *Theor. Appl. Genet.* 121:731-739

Shulaev V, et al. 2011. The genome of woodland strawberry (*Fragaria* vesca). *Nat. Genet.* 43(2):109-116

Sicard A, et al. 2011. Genetics, evolution, and adaptive significance of the selfing syndrome in the genus *Capsella*. *The Plant Cell* 23:3156-3171

Singh RJ, Hymowitz T. 1988. The genomic relationship between *Glycine max* (L.) Merr. and *G. soja* Sieb. and Zucc. as revealed by pachytene chromosome analysis. *Theor. Appl. Genet.* 76:705-711

Slotte T, et al. 2013. The *Capsella rubella* genome and the consequences of rapid mating system evolution. *Nat. Genet.* 45(7):831-5

Sondur SN, Manshardt RM, Stiles JI. 1996. A genetic linkage map of papaya based on randomly amplified polymorphic DNA markers. *Theor. Appl. Genet.* 93:547-553.

Song KM, Suzuki JY, Slocum MK, Williams PH, Osborn TC. 1991. A linkage map of *Brassica rapa* (syn. *campestris) based on restriction fragment length polymorphism loci. Theor. Appl. Genet.* 82:296-304

Song QJ, et al. 2004. A new integrated genetic linkage map of the soybean. *Theor. Appl. Genet.* 109:122-128

Spielmeyer W, Green AG, Bittisnich D, Mendham N, Lagudah ES. 1998. Identification of quantitative trait loci contributing to Fusarium wilt resistance on an AFLP linkage map of flax (*Linum usitatissimum*). *Theor. Appl. Genet.* 97:633-641

Sraphet S, et al. 2011. SSE and EST-SSR-based genetic linkage map of cassava (*Manihot esculenta* Crantz). *Theor. Appl. Genet.* 122:1161-1170

Sveinsson S, Gill N, Kane NC, Cronk Q. 2013. Transposon fingerprinting using low coverage whole genome shotgun sequencing in Cacao (*Theobroma cacao* L.) and related species. *BMC Genomics* 14:502

Swarbreck D, et al. 2008. The *Arabidopsis* Information Resource (TAIR): gene structure and function annotation. *Nuc. Acids Res.* 36:D1009-14.

Tang X, et al. 2009. Assignment of genetic linkage maps to diploid *Solanum tuberosum* pachytene chromosomes by BAC-FISH technology. *Chromosome Research* 17:899-915

Tanksley SD, et al. 1992. High density molecular linkage maps of the tomato and potato genomes. *Genetics* 132:1141-1160

Temnykh S, et al. 2000. Mapping and genome organization of microsatellite sequences in rice (*Oryza sativa* L.) *Theor. Appl. Genet.* 100:697-712

Teutonico RA, Osborn TC. 1994. Mapping of RFLP and qualitative trait loci in *Brassica rapa* and comparison to the linkage maps of *B. napus, B. oleracea,* and *Arabidopsis thaliana*. *Theor. Appl. Genet.* 89:885-894

The *Brassica rapa* Genome Sequencing Project Consortium. 2011. The genome of the mesopolyploid crop species *Brassica rapa. Nature Genetics* 43:1035–1039

The Potato Genome Sequencing Consortium. 2011. Genome sequence and analysis of the tuber crop potato. *Nature* 475(7355):189-195.

Thoquet P, et al. 2002. The molecular genetic linkage map of the model legume *Medicago truncatula*: an essential tool for comparative legume genomics and the isolation of agronomically important genes. *BMC Plant Biology* 2:http://www.biomedcentral.com/1471-2229/2/1

Tomato Genome Consortium. 2012. The tomato genome sequence provides insights into fleshy fruit evolution. *Nature* 485(7400):635-641

Troggio M, et al. 2007. A dense single-nucleotide polymorphism-based genetic linkage map of grapevine (*Vitis vinifera* L.) anchoring pinot noir bacterial artificial chromosome contigs. *Genetics* 176:2637-2650

Tuskan GA, et al. 2006. The geome of black cottonwood, *Populus trichocarpa* (Torr. & Gray). *Science* 313(5793):1596-604.

Tuskan GA, et al. 2004. Characterization of microsatellites revealed by genomic sequencing of *populus trichocarpa*. *Can. J. For. Res.* 34:85-93

Vallejos CE, Sakiyama NS, Chase CD. 1992. A molecular marker-based linkage map of *Phaseolus vulgaris* L. *Genetics* 131:733-740

Velasco R, et al. 2010. The genome to the domesticated apple (*Malus* x *domestica* Borkh.) *Nat. Genet.* 42(10):833-839

Verde I, et al. 2013. The high-quality draft genome of peach (*Prunus persica*) identifies unique patterns of genetic diversity, domestication, and genome evolution. *Nat. Genet.* 45(5):487-94.

Verhaegen D, Plomion C. 1996. Genetic mapping in *Eucalyptus urophylla* and *Eucalyptus grandis* using RAPD markers. *Genome* 39:1051-1061

Vuylsteke M, et al. 1999. Two high-density AFLP linkage maps of *Zea mays* L.: analysis of distribution of AFLP markers. *Theor. Appl. Genet.* 99:921-935

Wang ZM, Devos KM, Liu CJ, Wang RQ, Gale MD. 1998. Construction of RFLP-based maps of foxtail millet, *Setaria italica* (L.) P. Beauv. *Theor. Appl. Genet.* 96:31-36

Wang Z, et al. 2012. The genome of flax (*Linum usitatissimum*) assembled *de novo* from shotgun sequence reads. *Plant J.* 72(3):461-473

Wang G, et al. 2005. Construction of a cucumber genetic linkage map with SRAP markers and location of the genes for lateral branch traits. *Sci. China. C Life Sci.* 48(3)213-220

Wang K, et al. 2012. The draft genome of a diploid cotton *Gossypium raimondii*. *Nat. Genet.* 44:1098-1103

Wang W, Feng B, Xiao J, Xia Z, Zhou X, Li P, Zhang W, Wang Y, Moller BL, Zhang P, et al. 2014. Cassava genome from a wild ancestor to cultivated varieties. *Nature Communications* 5:5110

Weng Y, Johnson S, Staub JE, Huang S. 2010. An extended intervarietal microsatellite linkage map of cucumber. *Cucumis sativus* L. *Hort. Science* 45(6):882-886

Whelan EDP. 1969. Pachytene morphology of *Prunus avium* L. CV. Lambert. *Can. J. Genet. Cytol.* 11:125-132

Wu GA, Prochnik S, Jenkins J, Salse J, Hellsten U, Murat F, Perrier X, Ruiz M, Scalabrin S, Terol J, et al. 2014. Sequencing of diverse mandarin, pummelo and orange genomes reveals complex history of admixture during citrus deomestication. *Nature Biotechnology* 32:656-662

Xu G, Magill CW, Schertz KF, Hart GE. 1994. A RFLP linkage map of *Sorghum bicolor* (L.) Moench. *Theor. Appl. Genet.* 89:139-145

Xu Q, et al. 2013. The draft genome of sweet orange (*Citrus sinensis*). *Nat. Genet.* 45:59-66

Yamamoto T, Yamaguchi M, Hayashi T. 2005. An integrated genetic linkage map of peach by SSR, STS, AFLP, and RAPD. *J. Japan. Soc. Hort. Sci.* 74(3):204-213

Yamanaka N, et al. 2001. An informative linkage map of soybean reveals QTLs for flowering time, leaflet morphology and regions of segregation distortion. *DNA Research* 8:61-72

Yeboah MA, Xuehao C, Feng CR, Liang G, Gu M. 2007. A genetic linkage map of cucumber (*Cucumis sativus* L) combining SRAP and ISSR markers. *African Journal of Biotechnology* 6(24):2784-2791

Yogeeswaran K, et al. 2005. Comparative genome analyses of *Arabidopsis* spp.: Inferring chromosomal rearrangement events in the evolutionary history of *A. thaliana*. *Genome Res.* 15:505-515

Young ND, et al. 2011. The *Medicago* genome provides insight into the evolution of rhizobial symbioses. *Nature* 480(7378):520-524

Yuan XJ, et al. 2008. Genetic linkage map construction and location of QTLs for fruit-related traits in cucumber. *Plant Breeding* 127:180-188

Zhang W, et al. 2004. QTL mapping of ten agronomic traits on the soybean (*Glycine max* L. Merr.) genetic map and their association with EST markers. *Theor. Appl. Genet.* 108:1131-1139

Zhang X, Wessler SR. 2004. Genome-wide comparative analysis of the transposable elements in the related species *Arabidopsis thaliana* and *Brassica oleracea*. *PNAS* 101(15):5589-5594
