# Supplementary material for: The relationship of recombination rate, genome structure, and patterns of molecular evolution across angiosperms
Source: BMC Evol Biol. 2015 Sep 16;15:194. doi: 10.1186/s12862-015-0473-3 (PMC4574184; doi:10.1186/s12862-015-0473-3)
Supplement: Additional file 3: Figure S1. — Independent contrast plots of recombination rate vs genome compactness and chromosome number. Lines on each plot are the best-fit linear regression. The Pearson’scorrelation coefficient (r) and p-value (p) are given on each plot. Figure S2. Independent contrast plots of recombination rate vs GC content at 3rd position synonymous sites (3GCS) and codon bias (ENC). Lines on each plot are the best-fit linear regression. The Pearson’s correlation coefficient (r) and p-value (p) are given on each plot. (PDF 194 kb) [file 12862_2015_473_MOESM3_ESM.pdf]

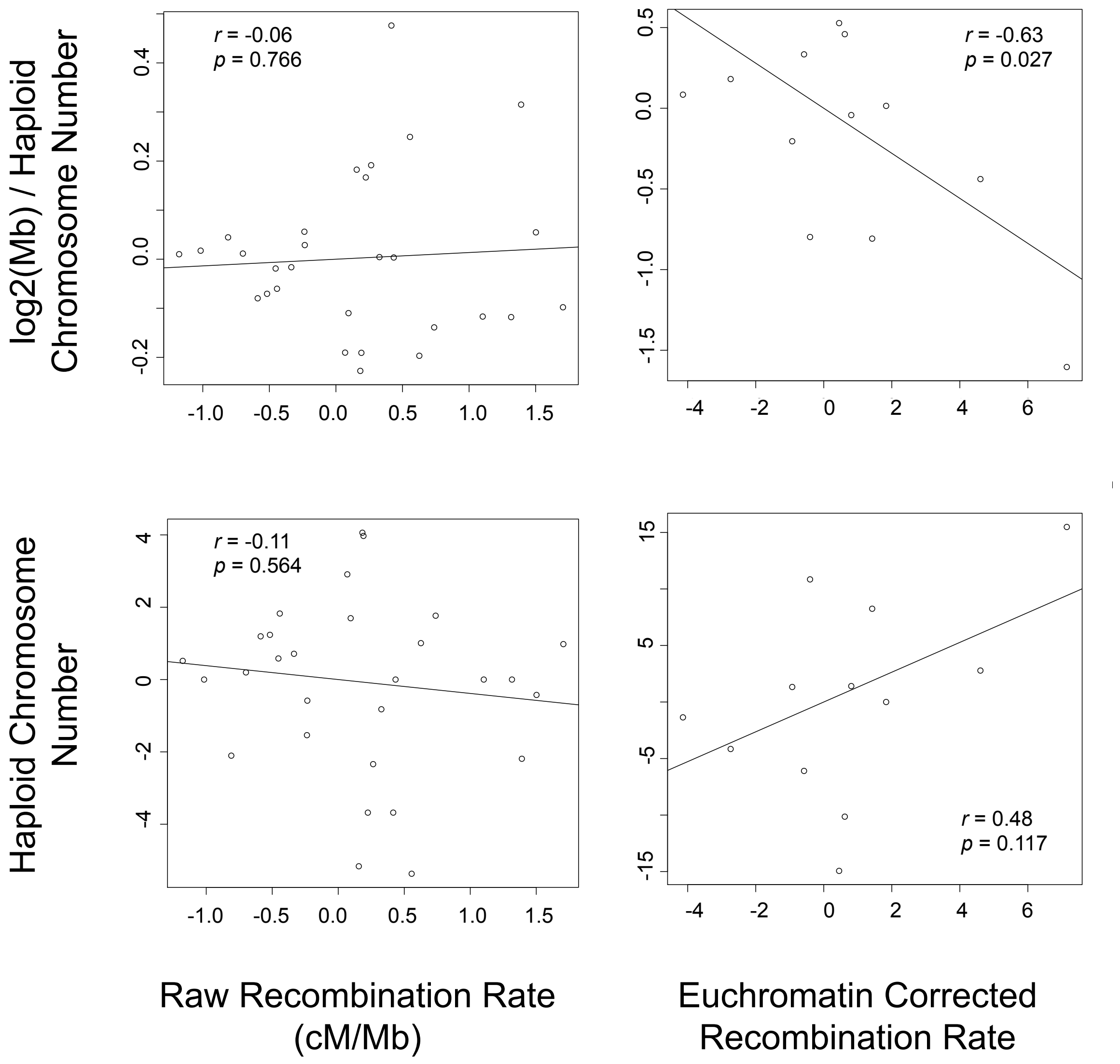

Figure S1 - Independent contrast plots of recombination rate vs genome compactness and chromosome number. Lines on each plot are the best-fit linear regression. The Pearson's correlation coefficient (*r*) and p-value (*p*) are given on each plot.

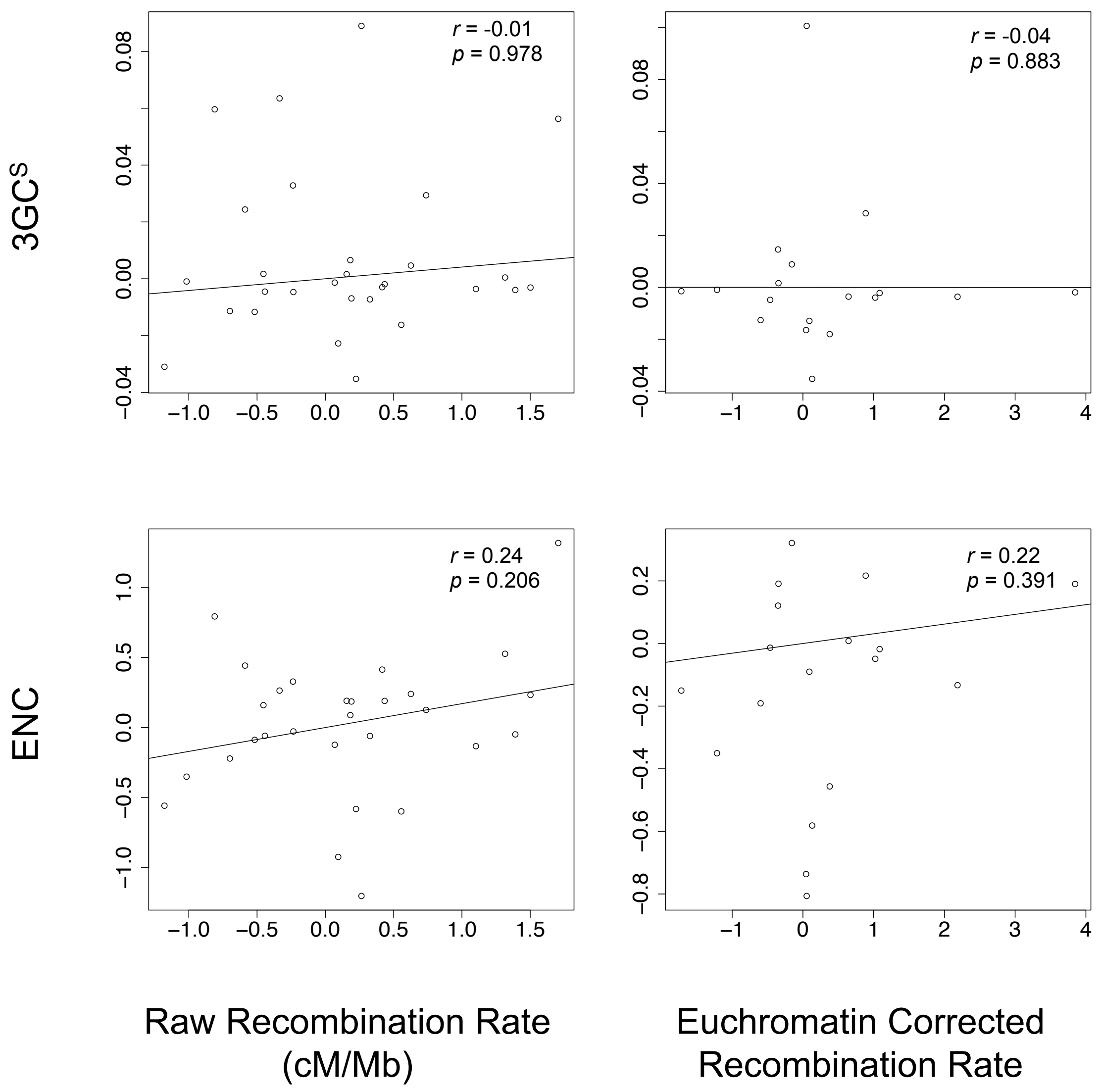

Figure S2 - Independent contrast plots of recombination rate vs GC content at 3rd position synonymous sites ($3GC^S$) and codon bias (ENC). Lines on each plot are the best-fit linear regression. The Pearson's correlation coefficient (*r*) and p-value (*p*) are given on each plot.
